# Bulk Crystallization in a SiO_2_/Al_2_O_3_/Y_2_O_3_/AlF_3_/B_2_O_3_/Na_2_O Glass: Fivefold Pseudo Symmetry due to Monoclinic Growth in a Glassy Matrix Containing Growth Barriers

**DOI:** 10.1038/srep19645

**Published:** 2016-01-27

**Authors:** Wolfgang Wisniewski, Martin Seyring, Christian Patzig, Thomas Höche, Ashkan Keshavarzi, Christian Rüssel

**Affiliations:** 1 Otto-Schott-Institut, Jena University, Fraunhoferstr. 6, 07743 Jena, Germany; 2 Fraunhofer Institute for Mechanics of Materials IWM, Walter-Huelse-Straße 1, 06108 Halle (Saale), Germany

## Abstract

A glass with the mol% composition 17 Y_2_O_3_·33 Al_2_O_3_·40 SiO_2_·2 AlF_3_·3 Na_2_O·2 CeF_3_·3 B_2_O_3_ is heat treated at 1000 °C for 6–24 h. This results in the surface nucleation and growth of YAG. Nucleation and growth of star-shaped alumina and later of monoclinic β-Y_2_Si_2_O_7_ and orthorhombic δ-Y_2_Si_2_O_7_ are additionally observed in the bulk. Phase identification and localization are performed by electron backscatter diffraction (EBSD) as well as TEM analysis. The monoclinic β-Y_2_Si_2_O_7_ observed in the bulk occurs in the form of large, crystal agglomerates which range from 50 to 120 μm in size. The individual crystals are aligned along the c-axis which is the fastest growing axis. Ten probability maxima are observed in the pole-figures illustrating the rotation of orientations around the c-axes indicating a fivefold symmetry. This symmetry is caused by multiple twinning which results in a high probability of specific orientation relationships with rotation angles of ~36°, ~108° (also referred to as the pentagon angle) and ~144° around the c-axis. All these rotation angles are close to the multiples of 36° which are required for an ideal fivefold symmetry. This is the first report of a fivefold symmetry triggered by the presence of barriers hindering crystal growth.

Five-fold symmetry in crystalline arrangements has been known for some decades. In principle, there are two possible reasons for a fivefold symmetry. Apart from the quasi crystals first reported by D. Shechtman in1984^1^ the other possibility to obtain a fivefold symmetry is via multiple twinning which was first observed in 1957 and reviewed extensively in 1998^2^. Here the alternating twins enclose angles of 72° to allow a five- or tenfold symmetry^2^,^3^. Fivefold twins were reported for a wide variety of materials^2^, most of which have a cubic symmetry such as nanocrystalline copper^4^, gold particles prepared by vapor condensation^5^ and diamonds prepared using a CVD process^6^. A strong tendency to multiple twins with fivefold symmetry has also been reported in monoclinic phases^7^,^8^, such as τ-AI_13_Co_4_, or τ-AI_13_Fe_4_. Here, (001), (100) as well as (20-1) glide twins are known to produce a 36° sector morphology. Fullerite (C_60_) structures reaching 2 mm in size have also synthesized^2^. These structures are usually proven using electron diffraction in a transmission electron microscope. Generally fivefold twins may occur as deformation or growth twins and according to some reports, high external stresses, as well as different stress orientations are a prerequisite for the formation of multiple twins^9^. Other reports based on molecular dynamics and some experimental studies conclude that external stresses are not necessary for the formation of fivefold twins in nano crystalline cubic materials^4^,^10^,^11^.

The above mentioned alloys are only examples and similar structures were also obtained from many other alloys with different chemical compositions. However, inorganic, non-metallic compounds other than boronsuboxide^12^, Fe_2_O_x_^13^, Ta_1.6_Te^13^,^14^ or diamond^6^ have not been reported to show a fivefold symmetry to the best of our knowledge.

Recently, the preparation of surface crystallized yttrium aluminum garnet (YAG) layers from a glass with the composition 17 Y_2_O_3_·33 Al_2_O_3_·40 SiO_2_·2 AlF_3_·3 Na_2_O·2 CeF_3_·3 B_2_O_3_ during thermal annealing was reported^15^ in the context of research concerning new phosphorous materials for LED-technology^16^. The YAG crystals were doped with Ce^3+^ and showed an intense green fluorescence when irradiated with UV-light^15^, see also Fig. 1. Simultaneous bulk nucleation led to the formation of Al-rich star-shaped crystals throughout the sample, depleting Al from the glass. The YAG layer only grew to a thickness of approximately 30 μm and was accompanied by the formation of pores at the growth front. These pores show an enhanced nucleation of the star-shaped phase at their surface. At some point, the nucleation of monoclinic ε-Y_2_Si_2_O_7_ and orthorhombic δ-Y_2_Si_2_O_7_ was triggered adjacent to the YAG layer blocking further YAG growth. Additionally, δ-Y_2_Si_2_O_7_ and a different monoclinic phase identified as β-Y_2_Si_2_O_7_ nucleated in the bulk^15^. All occurring yttrium silicates (YS) grew around the already existing star-shaped phase. While the previous article focused on the crystallization associated to the zone of surface crystallization, the aim of this article is to describe the growth structures formed by crystalline YS in the bulk of the glass. Special focus is given to the growth of β-Y_2_Si_2_O_7_ because the novel phenomenon of a five-fold symmetry induced by the presence of growth barriers is observed.

## Results and Discussion

**Figure 1:**
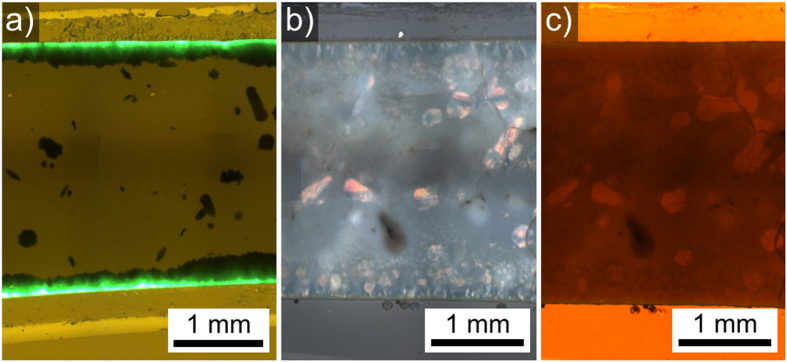
Optical microscopy of samples annealed at 1000 °C for (a) 6 h (fluorecence micrograph), (b) 24 h (polarization micrograph) (c) 24 h (transmitted light micrograph with an artificial red shift to enhance contrast.

The prepared glass was yellow and optically transparent. After crystallization the appearance of the samples ranged from yellow and translucent (6h) to white and opaque (24 h). Figure 1 presents optical micrographs of the cross sections of samples crystallized at 1000 °C. Fig. 1a) shows a fluorescence micrograph of a sample annealed for 6 h. The image was recorded using an UV-laser (wavelength 460 nm) resulting in strong green fluorescence at a wavelength of 530 nm (green) which is typical for Ce^3+^-doped YAG crystals^17^. The Fig. 1(b,c) show the same section of a sample annealed for 24 h: b) a polarization micrograph and c) a transmitted light micrograph in a false color illustration with an artificial red shift to enhance contrast. The zone of surface crystals is composed of three crystalline layers:(i) a fluorescent YAG layer immediately below the surface followed by (ii) a ε-YS layer (ICSD 28004) and (iii) a final layer of δ-YS (ICSD 33721)^15^. The large, dark structures in the bulk correspond to δ-YS and β-YS crystal agglomerates; they are the main focus of this article. The Fig. 1(b,c) are presented in order to illustrate the different optical properties of some crystal structures in these samples: the structures which appear colored in the polarization micrograph also show a higher transparency in the red-shifted light transmission micrograph. Additionally, the entire sample is filled with the star-shaped crystals of up to 10 μm diameter^15^.

**Figure 2:**
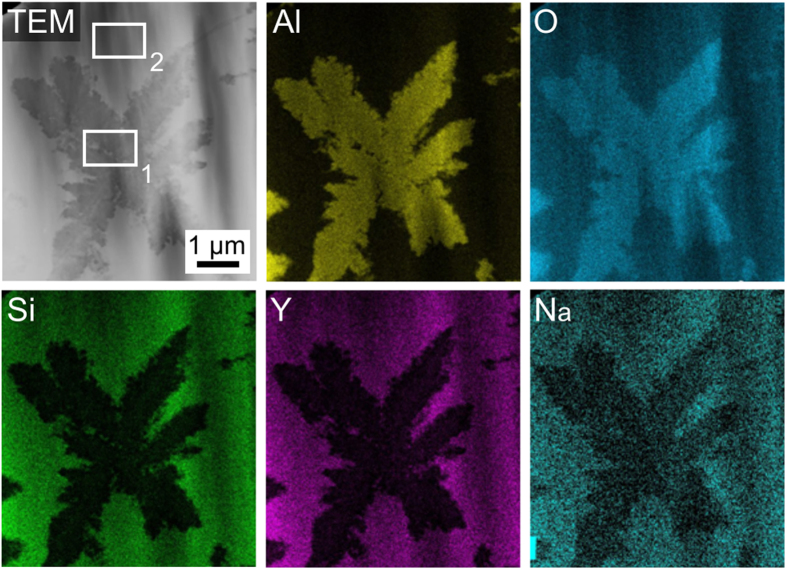
TEM EDX mapping of the star shape phase for the elements Al, O, Si, Y and Na.

This star-shaped phase could previously not be identified because it appeared amorphous to XRD-analyses and only enabled the acquisition of very diffuse EBSD-patterns which could not be indexed reliably^15^. These stars are so fine that a clear chemical analysis by EDX in the SEM is not possible due to the large information volume of the method^15^. TEM analyses of these stars were performed and led to the results presented in Fig. 2: Al and O are enriched while Si, Y and Na are depleted in the stars. Quantitative measurements were performed in the areas 1 and 2, proving that the star-shape phase has an Al:O ratio of about 2:3. Regarding the circumstances and the crystallization of corundum in a related melt^17^, it may hence be concluded that these stars are Al_2_O_3_ and most probably corundum. This phase is formed before the yttrium silicates nucleate in the bulk so that their growth is always confronted with a system of Al_2_O_3_ barriers.

**Figure 3:**
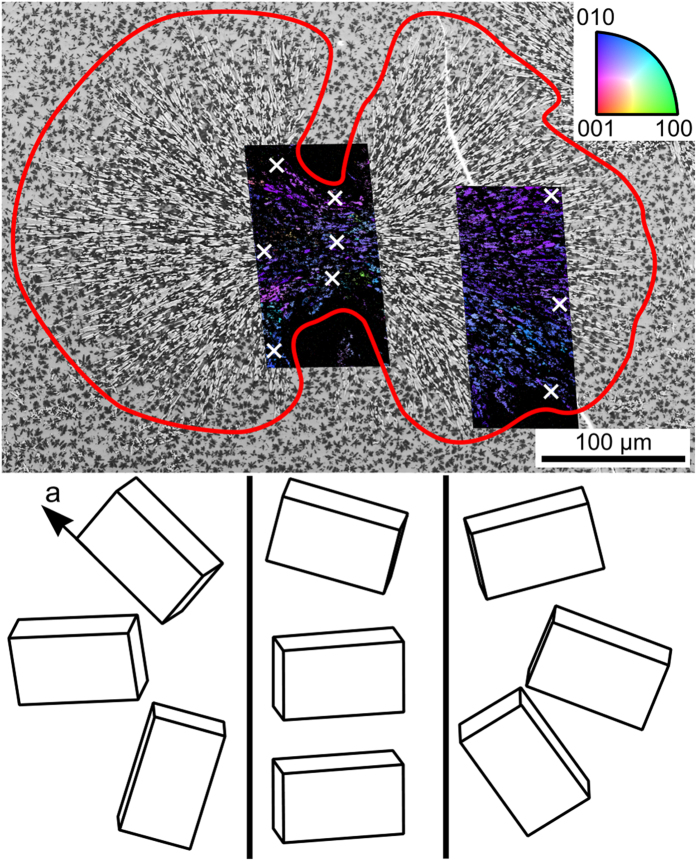
SEM-micrograph of a large δ- Y_2_Si_2_O_7_ growth structure framed in red and cut parallel to the a-axes of the crystals. The micrograph is superimposed by IPF + IQ-maps of performed EBSD-scans. The colors in the maps are attributed to the inverse pole-figure and hence indicate the orientation of the respective crystals. Bottom: Wire frames of unit cells visualize orientations of the crystals at the locations marked by white crosses.

In order to visualize the growth mechanism of the bulk structures, it is helpful to analyze specific cut planes along growth directions. Figure 3 presents the SEM-micrograph of a δ-YS growth structure unhindered by other YS-crystals during growth. The superimposed combined Inverse Pole Figure + Image Quality Maps (IPF + IQmaps) of performed EBSD scans already imply a non-statistical orientation distribution. The wire frames of δ-YS unit cells presented below visualize the crystal orientations at the positions marked by white crosses and confirm that the long a-axes of this phase are aligned to the streaks of crystal growth^15^. Assuming the center of nucleation were in the current cut plane, the longest distance perpendicular to the original a-axis is about 25 μm from the nucleation site while the longest distance parallel to the a-axis are more than 200 μm from the latter. Hence growth along the a-axis is about eight times as fast as in directions perpendicular to it in the given matrix. This structure was cut almost parallel to the a-axes of the crystals and clearly shows a notable change of the orientation: while the a-axes are rather parallel in the middle, they almost point in opposite directions at the extremes of the left-hand scan. While these structures imply spherulitic growth^18^, the spaces between the two halves are not crystallized at the current state.

**Figure 4:**
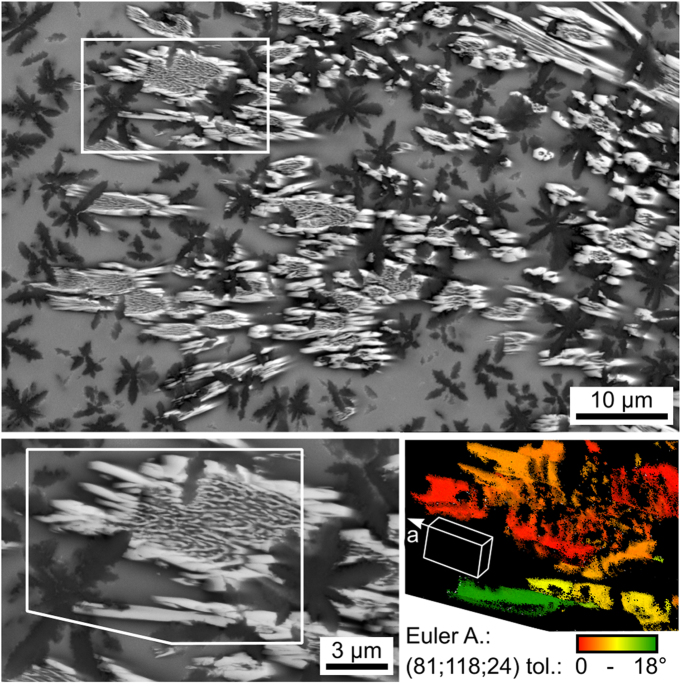
SEM-micrograph of a δ- Y_2_Si_2_O_7_ growth structure cut almost parallel to the a-axes of the crystals. Bottom left: the area framed above is presented with a higher magnification. Bottom right: orientation map of the area framed to the right. The crystal orientations change with the location according to the color scale. The presented unit cell visualizes the δ- YS orientation of the stated Euler Angle combination with a tolerance of 18°.

The individual channels of δ-YS grow in the form of the very fine crystals (bright) presented in Fig. 4 which necessitate a maximum step size of about 300 nm to visualize coherent crystals in EBSD-maps. The higher magnification shows that these channels do not always form compact crystals but rather areas of lamellar growth in a matrix surrounded by a compact crystalline ring. The presented orientation + IQ-map of the reliably indexed points in an EBSD-scan performed with a step size of 50 nm and cleaned using the CI-standardization shows that continuous orientation changes occur from red to yellow as well as the discrete change to green. The presented wire frame illustrates the orientation of the stated Euler Angle combination where the fast growing a-axis of the crystal is tilted from the cut plane by 24° (described by the Euler Angle φ_2_ = 24°). Immediate contact to the Al_2_O_3_-stars (dark) is observed as well as a high amount of uncrystallized glass (gray). It appears these crystals grew in the form of tubes filled by finer crystal structures. It may be concluded that δ-YS growth occurs in the form of spherulitic structures because after nucleation, growth mainly occurs in the direction of the a-axes. δ-YS grown in a glass free of the Al_2_O_3_-stars showed growth in the form of compact crystals^17^ instead of spherulitic growth, i.e. this mechanism is probably a result of the necessity to circumvent the Al_2_O_3_ during growth. Of course the different chemical conditions may also affect the growth morphology.

**Figure 5:**
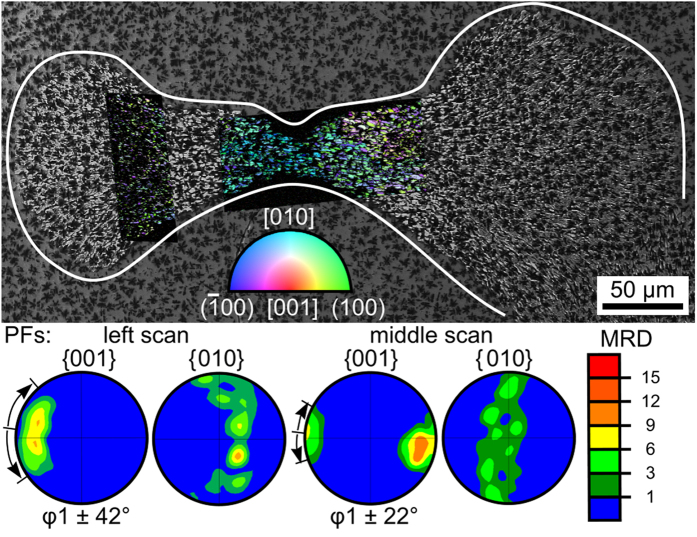
SEM-micrograph of a large β-Y_2_Si_2_O_7_ growth structure cut almost parallel to the c-axes of the crystals and framed in white. The superimposed IPF-maps indicate the orientation according to the color scale. Pole figures calculated from the scans performed in the left and middle parts of the growth structure are presented below.

Figure 5 presents a crystalline agglomerate resulting from β-YS growth in the bulk only hindered by the pre-existing Al_2_O_3_ stars. These growth structures are very different from those of δ-YS as they contain barely any residual glass within the crystallized area and show very different proportions. The presented pole figures (PFs) of the EBSD-scans performed in the areas superimposed by the IPF + IQ-maps show that the main direction of growth seems to be along the c-axes and the structure is again clearly textured. However, the orientation spread is much smaller compared to the δ-YS structure featured in Fig. 3: while the 001-PF of the scan in the middle indicates a spread of φ_1_ ± 22°, the 001-PF of the left hand scan indicates a spread of φ_1_ ± 42°. In these PFs, the Euler Angle φ_1_ describes the rotation of the c-axis around the surface normal. The 010-PFs also show orientational preferences in the rotation around the c-axis. The presented probability legend is valid for all subsequent PFs of textures.

**Figure 6:**
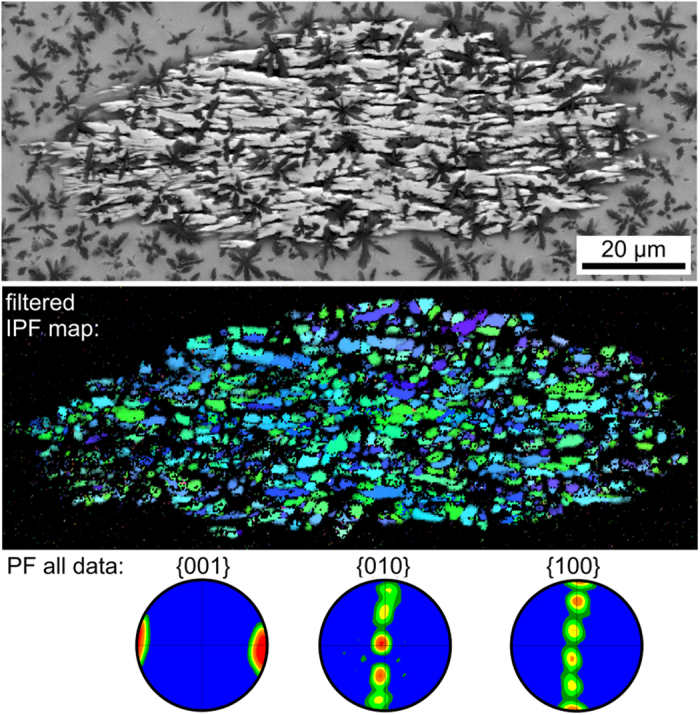
Top: SEM-micrograph of a small β-Y_2_Si_2_O_7_ growth structure cut parallel to the c-axes of the crystals. Bottom: IPF-map of an EBSD-scan performed on the area and pole-figures of the scan showing the five fold symmetry via five preferred orientations. The IPF-scale presented in Fig. 5 also applies here.

These preferences are much clearer in Fig. 6 which presents comparable data of an early stage of β-YS growth with the c-axes truly parallel to the current cut plane (see the 001-PF). The presented 010- and 100-PFs show that the a- and b-axes preferably assume five orientations in rotation around the c-axis. The positions of high probability in the 010- and 100- PFs vary alternately: if the probability is high in the 010-PF, it is low in the 100-PF and vice versa. This is logical considering that the poles of the a- and b- axes show a 36° orientation difference to each other. In the monoclinic unit cell, the a- and b-axes enclose the angle γ = 90° meaning that the (100)-pole of every (010)-pole is rotated by 90° and hence positioned between the {100} poles at 72° and 108°.

**Figure 7:**
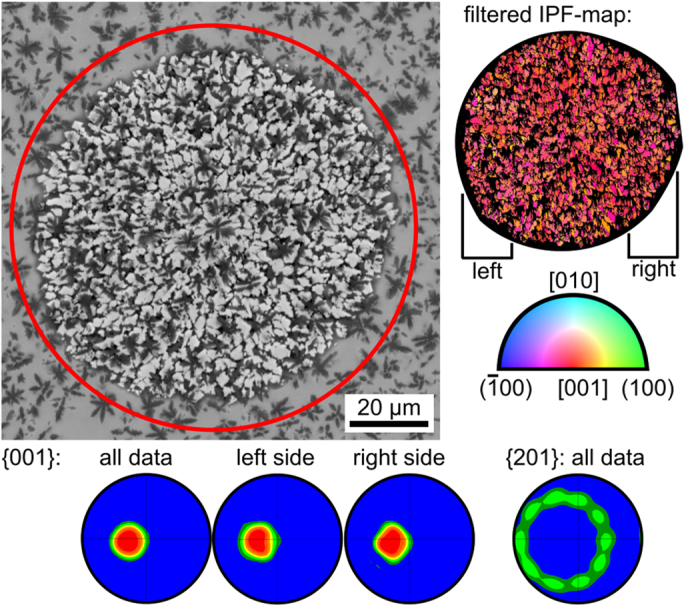
SEM-micrograph where the β-Y_2_Si_2_O_7_ growth structure is framed in red. To the right: IPF + IQ-map of an EBSD scan performed on this area. Bottom: pole figures of the EBSD-scan. The ten maxima in the 201-PF are all arranged on a circle giving evidence of the five fold symmetry.

Comparable results of a β-Y_2_Si_2_O_7_ growth structure cut almost perpendicular to the c-axes of its crystals are presented in Fig. 7: the spherical shape of the crystallized area in the SEM-micrograph shows that growth velocities perpendicular to the main direction of growth are homogeneous. The presented IPF + IQ-map clearly indicates a texture and the presented 001-PFs show that these textures do not vary between the left and right part of the scan. The 001-PFs illustrate that the c-axes are tilted by about 11° from the surface normal of the cut plane. The 201-PF is presented because it is well suited to illustrate the non-statistical rotation around the c-axis: each preferred orientation leads to two opposite probability peaks, hence the ten peaks in the PF confirm the five-fold symmetry already indicated in Fig. 6. As this symmetry is not discrete but subject to a certain orientation tolerance it is subsequently referred to as a pseudo symmetry.

**Figure 8:**
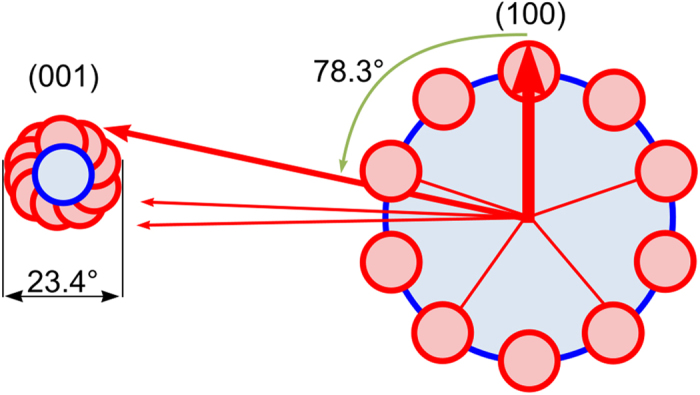
Schematic illustration of the relationship between the 001- and 100-pole figures.

Furthermore, the (001)-PFs show a much broader frequency distribution of the (001) plane normal from the mean value compared to the approximately discrete distributions in other PFs. The texture presented in Fig. 7 is schematically illustrated in Fig. 8. Due to the monoclinic symmetry, the [001] -direction is not perpendicular to the (001)-plane spanned by the [100]- and [010]-directions. This indicates the rotation axis of orientation change is not the (001) pole but the c-axis of the unit cell. Due to the monoclinic angle of β = 101.7° the (100) and the (001) poles include an angle of 78.3°.The crystals preferably assume orientations separated by 36° of rotation around the c-axis which is exactly perpendicular to all (100) poles. Thus, this rotation is accompanied by a precession of (001) poles around the c-axis with an apex angle of 23.4°, which is in agreement with the spread of the 001-pole of about 22° (see the middle scan of Fig. 5).

**Figure 9:**
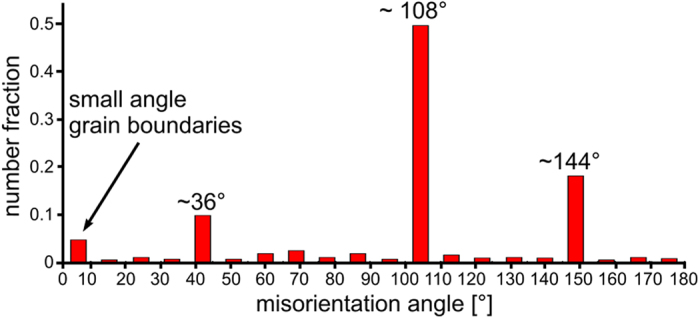
Misorientation angle distribution and corresponding misorientation axes of the β-Y_2_Si_2_O_7_ growth structure displayed in Fig. 7. This is clear evidence of the preference of specific angles, which all are in agreement with a five fold symmetry due to multiple twinning.

Figure 9 represents the misorientation angle distribution and the distribution of the corresponding misorientation axes of the β-YS growth structure displayed in Fig. 7. The orientation relationship of two adjacent grains is described by the misorientation which is defined by a misorientation angle and a misorientation axis^19^. The misorientation angle distribution proves the presence of high fractions of special orientation relationships. There is a high frequency of small-angle grain boundaries and misorientations with angles of about 36°, 108° and 144°. The distribution of the corresponding misorientation axes (below) reveals that the most of these special orientation relationships possess a misorientation axis parallel to [001]. It is obvious that these special orientation relationships are related to the five-fold symmetry of the crystallographic texture: On the one hand, the misorientation angles which are multiples of 36°, including the pentagon angle of 108°, produce the fivefold texture, whereas on the other hand the misorientation axes parallel to [001] establish the c-axis as the axis of the fivefold symmetry. Thus, all pole figures of directions that deviate from [001] exhibit this fivefold symmetry i.e. [100], [010] and [201] as displayed in the Figs 5, 6, 7.

**Figure 10:**
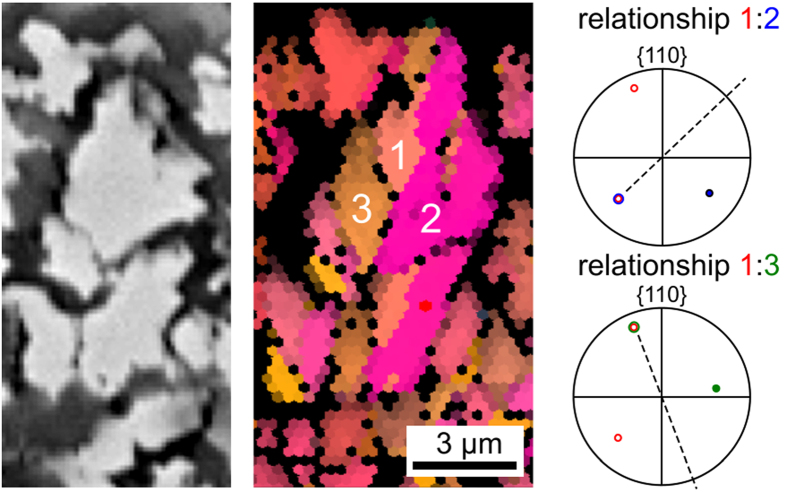
Right: SEM micrograph of some adjacent β-Y_2_Si_2_O_7_ crystals from the structure featured in Fig. 7. Middle: IPF + IQ-map of the same area. Right: poles of two neighboring crystals give the relationships between neighboring areas of orientation; misorientation angles: 1–2 = 106°, 1–3 = 107°, 2–3 = 147°.

Now the question arises why the ~108°angle between two β-YS grains occurs so often. Figure 10 presents the SEM-micrograph and the corresponding IPF + IQ-map segment of part of the structure presented in Fig. 7. While no clear grain boundaries may be discerned in the SEM-micrograph, the crystal lattices 1–3 clearly have different orientations. Their misorientation relationships exhibit the [001]-direction as the rotation axis and the misorientation angles are 106° (1–2) and 107° (1–3), i.e. very close to the pentagon angle of 108° which exhibits the highest frequency in the misorientation angle distribution presented in Fig. 9. The position of the {110}-planes of the neighboring crystals are illustrated by the respective PFs where circles represent points in the negative hemisphere. Both relationships share a parallel (110)-plane while the other (110)-planes have positions apparently mirrored across the PF-center, indicating a possible twinning relationship. In both cases the alignment of the mirror plane (dashed line) is almost parallel to the straight trace of the corresponding grain boundary in the IPF + IQ-map. Additionally, the parallel (110)-poles are intersected by the mirror plane, suggesting coherent boundaries which establish a mirror relationship, a feature characteristic for twin boundaries. The lattices 2 and 3 include a misorientation angle of 147°. This relationship does not show a coincidence in any low indexed PF. However, this misorientation angle can be seen as a result of the two other misorientation angles and the necessity for crystallographic consistency, i.e. all three misorientation angles should sum up to 106° + 107° + 147° = 360°. According to the misorientation angle distribution in Fig. 9 these orientation relationships are representative and correlated to the textures described throughout the EBSD-scans presented in Figs 6 and 7.

**Figure 11:**
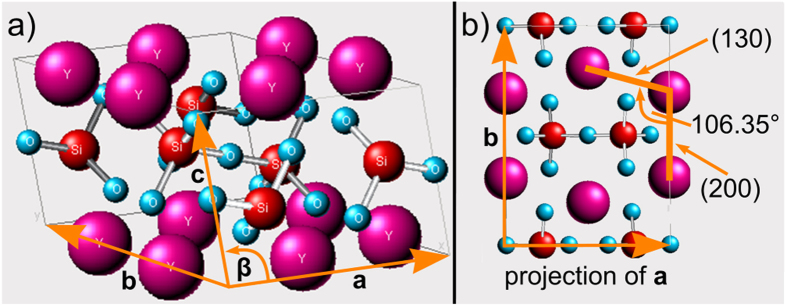
(**a**) Crystal structure of Y_2_Si_2_O_7_ and (**b**) unit cell in [001]- projection.

The unit cell of β-YS is displayed in Fig. 11a) to illustrate the crystallographic configuration of such a straight β-YS grain boundary with a 108° misorientation. The lattice constants of the crystal as taken from literature are a = 6.8691 Å, b = 8.9602 Å and c = 4.7168 Å with β = 101.73°(ICSD 281312). Yttrium (magenta) has a coordination number of six, oxygen (blue) two or three and silicon (red) four. One SiO_4_-tetrahedron is connected to another SiO_4_-tetrahedron via an Si-O-Si bridge and three further oxygen atoms which are each connected to two yttrium atoms. The latter oxygen atoms have a coordination number of three. Figure 11(b) displays the crystal structure in [001]-orientation. The view along the c-axis of the lattice produces a projection of the atom columns. The angle included by the (130) and the (200) plane is about 106.4° which is very close to the pentagon angle of 108°. Indeed this arrangement of the atom columns establishes the five-fold pseudo symmetry.

**Figure 12:**
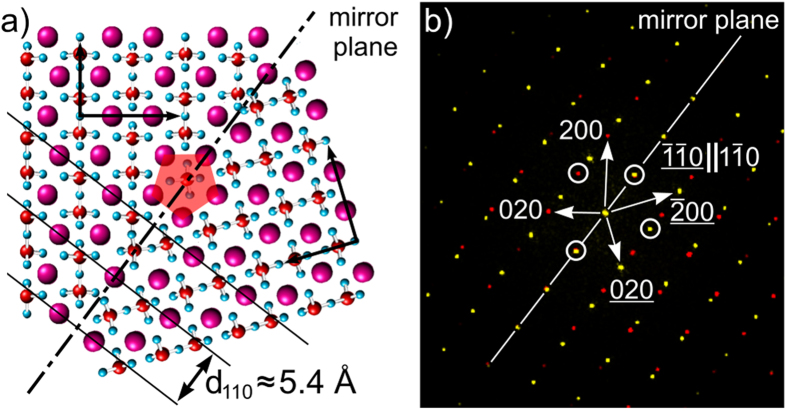
(**a**) two Y_2_Si_2_O_7_ crystal lattices rotated 108° to each other forming a twin boundary with a coherence of the (110) lattice planes; (**b**) corresponding diffractogram displaying the mirror symmetry and coincidence of several reflections.

The misorientation angle distribution in Fig. 9 shows that the 108° rotation is the most probable relationship between adjoining β-YS crystals. Figure 10 suggests that such crystals form a twin relationship with a coherent twin boundary perpendicular to the (110)-planes and parallel to the [001]-direction. Figure 12(a) illustrates a possible configuration of such a coherent 108° twin boundary. The twin boundary indicated by the dashed line acts as the mirror plane resulting in coherent (110) planes through both crystals (black lines).The corresponding diffractogram in Fig. 12(b) displays the twin relationship in terms of the reciprocal lattice. The encircled {110} reflections show the same characteristics as in the 110 PFs in Fig. 10: the reflections are coincident or show a mirror symmetry with a mirror plane (dashed line) that intersects the coincident reflections.

**Figure 13:**
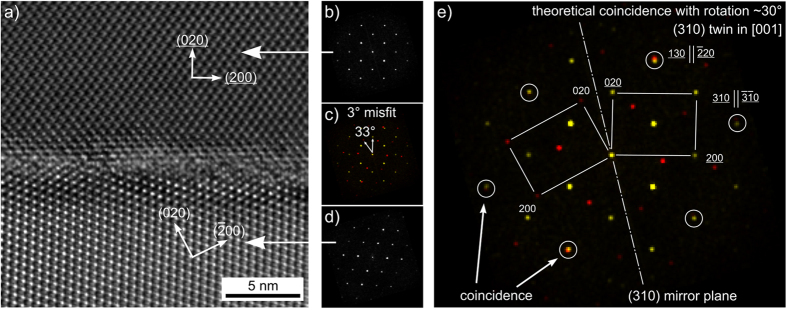
TEM observation of a grain boundary with a misorientation of 33°. HRTEM image with corresponding FFT diffractograms (**a**–**d**) compared to an ideal twin relationship based on a rotation of 30° (**e**).

In an attempt to confirm this relationship, FIB-lamellae were cut from β-YS growth structures and prepared for TEM analysis. The higher resolution in electron diffraction gained from the latter technique^19^, and in particular elemental mapping using the SuperX detector, has proven extremely beneficial for getting deeper insights into microstructure formation mechanisms in glass ceramics^20^,^21^,^22^,^23^. A grain boundary with a misorientation angle of about 33° is presented in the high-resolution micrograph Fig. 13a). The orientations of the two adjacent grains were determined by Fast Fourier Transformation (FFT) diffractograms which are presented in Fig. 13 (b, d). Figure 13(c) shows an overlay of the two diffractograms to visualize their misorientation. The specific reflections of each grain are displayed in yellow(upper grain) and red (lower grain).The misorientation angle of 33° is fairly close to the 36° which would present the ideal twin relationship. However, the optimal configuration illustrated in Fig. 13(e) is at a misorientation angle of 30° which exhibits several coincidence reflections (circled in white) where the (310)-plane serves as the mirror plane.

**Figure 14:**
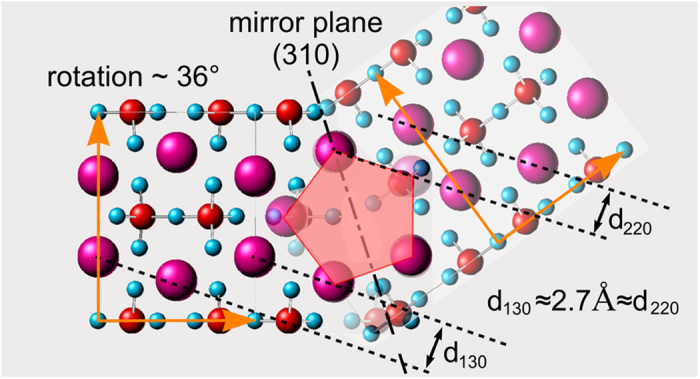
Two Y_2_Si_2_O_7_ crystals mirrored at the (310) plane. The rotation of 36° establishes a 5-fold symmetry as indicated by the red pentagon.

An atomistic model of the grain boundary analyzed of Fig. 13 with a misorientation angle close to 36° is presented in Fig. 14. Due to mirroring at the (310)-plane, a five-fold symmetry is possible as indicated by the red pentagon. Furthermore, the coincidence of the (130) and (220) reflections (compare Fig. 13(e) is explained, because the (130) and (220)-planes are aligned parallel and almost share the same interplanar spacing.

However, this 36° rotation does not produce a highly coherent boundary as seen in the HRTEM micrograph of Fig. 13(a). Furthermore, the observed alignment of the grain boundary plane appears to be random, as it does not correspond to the (310) mirror plane.

Compared to the 108° misorientation, the 36° and 144° misorientations show a low degree of coherence at their boundaries and are thus less favorable types of grain boundaries because they do not offer low interfacial energies^27^.The high probability of 36° and 144°misorientation angles could result from the pseudo symmetry, i.e. they occur when different 108° twins grow together as e.g. observed in Fig. 10. This is a reasonable explanation for the relationship of multiple twinning in this system and the observed five-fold symmetry.

The FFT diffractograms calculated from high resolution TEM images, e.g. Fig. 13 a), are not in precise agreement with a comparable pattern simulated using the literature values of the lattice constants. While the a/b ratio of the lattice constants in the literature is 0.7666, the ratio of the lattice constants determined from the FFT diffractograms is 0.7609, i.e. a deviation ca. 1%. One explanation for this deviation could be inherent stresses due to volumetric changes during crystallization, but it is also quite probable that Ce^3+^ is incorporated into the β-YS lattice, causing somewhat different lattice constants.

As reported in the refs 25 and 26, Phase Field Simulations have shown that the growth of single crystals and dendrites can be perturbed by the occurrence of heterogeneities. This model was originally derived for thin films of surface modified clay suspended in polymers. It was shown that the growth direction may change whenever a crystal reached a clay particle (≈1 μm in size)^25^. In other words, the model describes crystal growth in the presence of obstacles disturbing growth. While a particle may be pushed away at low growth velocities, the free enthalpy can most effectively be minimized by forming new grains or by changing the orientation and /or growth direction at high growth velocities. The result of both simulations and experiments is a polycrystalline structure caused by a sequential deflection of the crystal tips^25^. The extent to which deflection occurs, i.e. the angle of direction change, is arbitrary according to these simulations and does not assume systematic values.

In the system presented here, the β-YS crystals grow in a matrix of highly viscous glass filled with star-shaped Al_2_O_3_ crystals which reach a size of 6 μm before the bulk nucleation of the β-YS is initiated and finally grow up to 10 μm^15^. The β-YS grains have a size of 2 to 10 μm along the c-axis and about 1 to 5 μm perpendicular to it. That means the grains are elongated in the fastest growing direction. The crystals grow until they reach an Al_2_O_3_-crystal and then change their direction.

In summary it may be stated that δ-Y_2_Si_2_O_7_ nucleates in the bulk to form large, spherulitic structures composed of δ-Y_2_Si_2_O_7_-tubes surrounding lamellar δ-Y_2_Si_2_O_7_ embedded in an amorphous matrix of residual glass. δ-Y_2_Si_2_O_7_ growth mainly occurs along the crystallographic a-axis and the observed orientation spread and spherulitic morphology are most probably a result of crystal growth in a matrix filled with Al_2_O_3_-stars.

β-Y_2_Si_2_O_7_ is solely found in the bulk and also forms large, more compact growth structures which show a five-fold pseudo symmetry in texture analysis due to repeated twinning. Of the three possible formation mechanisms proposed for five-fold symmetries^2^ successive growth twinning seems most probable for the currently discussed system as there is no systematic layer by layer growth or deformation energy in the glass-ceramic. As with the spherulite like growth of δ-YS, the five-fold pseudo symmetry detected in the β-YS growth structures is most probably a result of crystal growth in a matrix filled with barriers in the form of the Al_2_O_3_-stars. β-YS grown in a related glass free of these barriers^17^ shows compact crystal bodies and even twinning probably as a mechanism of stress relaxation. It seems probable that the twins observed in this glass ceramic are independent of external stresses but are instead part of the grain growth and hence different from the sequential twinning process described in nanocrystalline Cu^4^.The structures showing fivefold pseudo symmetries here reach more than 100 μm in diameter in contrast to the nm-scale where these effects are usually observed^4^.

## Methods

A glass with the mol% composition 17 Y_2_O_3_·33 Al_2_O_3_·40 SiO_2_·2 AlF_3_·3 Na_2_O·2 CeF_3_·3 B_2_O_3_ was prepared from reagent grade raw materials. It was melted in a batch of 100 g in a platinum–rhodium crucible using an electric furnace heated to 1590 °C for 3 h. During melting, the melt was stirred manually from time to time using a platinum rod in order to homogenize the melt. The melt was cast on a copper block, quenched with a copper stamp and subsequently transferred to a furnace preheated to 850 °C. The furnace was switched off, allowing the samples to cool to room temperature with a rate of approximately 4 K/min.

Glass samples were cut, ground, polished and finally rinsed with ethanol and deionized water to remove any dust or polishing media and so prevent impurity induced surface crystallization. The samples were crystallized at a temperature of 1000 °C using a heating rate of 10 K/min. The annealing temperature was kept for of to 24 h before the samples were cooled to 830 °C using a rate of 4 K/min. The furnace was finally switched off, allowing the samples to cool to room temperature.

Fluorescence optical micrographs (FM) were obtained via laser scanning microscopy (LSM) using a Carl Zeiss Axio Imager Z1M LSM5-Pascal. Scanning electron microscopy was performed using a Jeol JSM-7001F scanning electron microscope (SEM) equipped with an EDAX Trident analyzing system containing a TSL Digiview3 EBSD camera. Electron Backscatter Diffraction (EBSD) was accomplished in the SEM. The EBSD scans were captured and evaluated using the programs TSL OIM Data Collection 5.31 and TSL OIM Analysis 6.2. Pole figures of textures are presented in multiples of a random distribution (MRD). The scans were acquired using a current of about 2.40 nA (measured with a Faraday cup) and a voltage of 20 kV.

Microstructure characterization down to the sub nanometer level using transmission electron microscopy (TEM) requires the preparation of electron-transparent sections. Two differently prepared samples were investigated for the studies presented here. From the bulk, a thin section was obtained by purely mechanical wedge polishing using the Allied MultiPrep System TEM wedge polisher. Prior to TEM investigation, this non-conducting sample had to be selectively carbon-coated using the CoatMaster^28^ in order to avoid static charging under electron irradiation. Moreover and in order to be able to analyze a specific sample area with TEM and EDXS, a target preparation of the TEM sample was performed by means of a focused ion beam (FIB) – cut of a TEM lamella using a focused Ar^+^ ion beam at 30 keV (Crossbeam NVision 40, Zeiss company), followed by a subsequent fine-polishing of the lamella with Ar^+^ ions at 5 keV.

TEM analyses were performed using a TITAN^3^G2 80-300 electron microscope at an acceleration voltage of 300 kV (FEI Company). Scanning TEM analysis in combination with energy dispersive X-Ray spectroscopy (EDXS) was performed on the same microscope using a high-angle annular dark field (HAADF) detector (FischioneModel 3000) and a Super-X EDX detector consisting of four silicon drift detectors, (FEI Company). The EDXS data analysis was done using the commercially available software Esprit (Bruker Company).

## Additional Information

**How to cite this article**: Wisniewski, W. *et al.* Bulk Crystallization in a SiO_2_/Al_2_O_3_/Y_2_O_3_/AlF_3_/B_2_O_3_/Na_2_O Glass: Fivefold Pseudo Symmetry due to Monoclinic Growth in a Glassy Matrix Containing Growth Barriers. *Sci. Rep.* **6**, 19645; doi: 10.1038/srep19645 (2016).
